# Challenges in assessing the clinical utility and economic value of immune checkpoint inhibitor therapies of Cancer

**DOI:** 10.1186/s40425-019-0707-9

**Published:** 2019-09-03

**Authors:** Peter Paul Yu, Omar Eton, Louis P. Garrison

**Affiliations:** 1 Hartford HealthCare Cancer Institute and Memorial Sloan Kettering Cancer Center, Hartford Hospital, 80 Seymour Street, Hartford, CT 06102-5037 USA; 2 Hartford HealthCare Cancer Institute, 80 Seymour Street, Hartford, CT 06102-5037 USA; 3 The Comparative Health Outcomes, Policy, and Economics (CHOICE) Institute, University of Washington, Health Sciences Building, H375, 1959 NE Pacific Street, Box 357630, Seattle, WA 98195-7630 USA

**Keywords:** QALY, Immune checkpoint inhibitors, Cost-effectiveness

## Abstract

Advances in the immunotherapy of cancer have prolonged survival for cancer patients, but the clinical and financial impact of treatments must be considered in determining the overall clinical utility and economic value of therapeutic agents. Quality-adjusted life years and incremental cost-effectiveness ratios are clinical and economic metrics that can be used to evaluate the value of immune checkpoint inhibitors. This Commentary provides perspective on the limitations, benefits, and potential enhancement of this approach to support value-based medicine.

The first two decades of the twenty-first Century have yielded truly substantive advances in our understanding of: a) the impact of driver mutations in individual patients’ cancers; and b) the dynamic relationship between tumors and the host’s ability to mount an effective anti-tumor immune response. In 2005, the number of oncology drugs in clinical development was 359 compared to 586 in 2015, an increase of 63%. From 2011 to 2015, 70 new cancer drugs were approved but with a significant concomitant increase in healthcare costs [1]. An analysis of all oncology drugs receiving Food and Drug Administration (FDA) approval between 2009 and 2013 found that the median launch price for a course of treatment was $116,100 for drugs with a novel mechanism of action and $119,765 for a drug within an established class of agents [2]. Data from the CMS Oncology Care Model for the period January 2014–June 2015 showed that oncologic drugs accounted for 39.1% of the total cost of care associated with episodes of chemotherapy, a percentage likely to increase and become an increasing threat to economic sustainability [3]. In 2018, the President’s Cancer Panel report focused on the rising cost of cancer drugs, their negative impact on patients and the need to relate drug price to the value provided [4].

Value assessment is derived from benefits and costs viewed through clinical, economic, and patient experience domains. Clinical value or utility is the measure of increase in quantity of life (survival) while accounting for impacts on quality of life (QoL)—either decrements due to treatment-related adverse events (AEs) or disease progression, or improvements in functioning and well-being due to disease response. The American Society of Clinical Oncology (ASCO) and the European Society for Medical Oncology (ESMO) have both proposed frameworks to assess the clinical utility of cancer therapeutics and these two tools were found to have moderate concordance when evaluated against 97 clinical trials [5]. The value assessments are based on algorithms incorporating survival, quality of life, toxicity and long term survival. Cost is not directly factored into the frameworks, but is considered in three other value frameworks; Memorial Sloan Kettering Drug Abacus [6], National Comprehensive Cancer Network Evidence Blocks [7] and the Institute for Clinical and Economic Review (I.C.E.R.) [8] The value frameworks of both ASCO [9] and I.C.E.R. refer to clinical utility as “net health benefit” (NHB) although they differ in definition. I.C.E.R. employs a more quantitative, event-specific model for toxicity assessment as described below. Further, the ASCO value-based framework is constrained to comparisons within a randomized clinical trial and its primary aim is use in shared decision-making between patients and providers, while I.C.E.R. evaluates each treatment’s benefit independently but in a manner that enables comparison among different treatments using a healthcare sector and societal perspective.

I.C.E.R defines economic value as the cost to deliver a unit of NHB expressed as the incremental cost-effectiveness ratio (ICER), with net impact on costs (in the numerator) and NHB (in the denominator). These measures can be evaluated a) from different perspectives—societal, health plan, and patient—and b) in different decision contexts—health plan coverage/formulary inclusion, development of clinical guidelines and pathways, and shared clinical decision-making at the patient level [10]. The latter should reflect individual patient’s personal preferences and beliefs as well as the out-of-pocket costs they face.

Verma et al. [11] report a systematic review of modeling projections of ICER for immune checkpoint inhibitors (ICI) in four different cancers. The models project the likely ICER over some time horizon based on clinical trial data and mathematical extrapolation of the presumed survival benefit beyond the observed trial data. Furthermore, the models attempt to take into account the quality of life experienced by surviving patients. Thus, the NHB is expressed in terms of the difference in “quality-adjusted life years” (QALY). The QALY measures survival while taking into consideration disease state and AEs. A gain of 1 year in survival is valued at something less than 12 months depending on the impact of disease and AEs on QoL. The ICI models on which these ICER estimates are based are often complex synthetic mathematical models (often Markov health-state transition models) that project overall survival and time spent in disease states of stable disease (SD) and progression of disease (PD), with some models adding time in clinical response. Each disease state is assigned a numeric value called a utility score which is less than 1 (the assumed utility value for perfect health). Immunotherapy-specific criteria of response have been developed (iRECIST) which redefine assignment of disease states [12]. ICI has been associated with longer SD durations than typically seen with chemotherapy, even off therapy, suggesting that some patients with radiographically determined SD may be disease free, and if this is associated with clinical benefit a higher utility value during this prolonged SD may be justified compared to that assigned to chemotherapy associated stable disease [13].

In addition, AEs are assigned negative values called disutilities which are subtracted to arrive at the net QALY. Ideally, the clinical trial on which the ICER is based would have captured the incidence, severity, and duration of AEs utilizing standardized instruments such as the NCI CTCAE v5.0 [14] and QoL assessment tools. Patient-reported outcomes (PRO) instruments (such as the EuroQoL EQ-5D) can be used to estimate health-related quality of life for study patients. A review of PRO measurements reported in case report forms within the FDA database of ICI registration trials for the first 5 ICI found that 75% of 28 registry trials included at least two PRO instruments. The two most commonly used instruments were EQ-5D in 90% of trials, and the European Organization for Research and Treatment of Cancer Quality of Life Questionnaire (EORTC QLQ-C30) which was reported in 81% of the trials. The review identified 8 ICI associated adverse events with frequencies exceeding 20% and evaluated the capture of these in the PRO instruments used in the registry trials. None of the instruments included AEs of rash or pruritus, and the median number of the 8 adverse events reported was only 3 [15]. Although FDA policy permits the use of PRO in considering regulatory approval, there is no current requirement to include PRO in registry studies.

The amount that AEs reduce a life-year gained is calculated by applying disutility weights (or “utility decrements”) to the trial-based clinical epidemiological parameters of AE incidence, severity, and duration. The studies vary in the utility values attributed to disease states and the disutility values attributed to AEs as seen in Table 1. The values for all studies were obtained from published literature, which are not specific to ICI. Disutility values derived from the literature generally reflect chemotherapy-related AEs. However, some AEs—e.g., pneumonitis or diarrhea—have significantly different clinical impact when a consequence of immunotherapy rather than chemotherapy and disutility values that account for the unique attributes of ICI are not available.

**Table 1:** Summary of Key Utility Parameter Estimates in Studies Cited in the Systematic Review

Author	QoL Source	PF Utility	PD Utility	Pulmonary Disutility	Diarrhea Disutility	Endocrine Disutility
Ward	CheckMate 141 EQ-5D QLQ-C30 QLQ-H&N 35	0.680	0.660	NR	NR	NR
Zagar	CheckMate 141	NR	NR	−0.200 IO	−0.207 IO	−0.210 IO
Tringale	CheckMate 141	0.517	0.280	NR	NR	NR
Goeree	CheckMate 017 EQ-5D	0.789	0.674	-0.008	NR	NR
Matter Walstra	Literature	0.756	0.470	NR	NR	NR
Aguilar	Literature	0.65	0.43	NR	NR	NR
Huang	KEYNOTE 010 EQ-5D	0.761	0.687	−0.09	NR	NR
Huang	KEYNOTE 024 EQ-5D	NR	NR	NR	NR	NR
Wan	CheckMate 025 EQ-5D	0.848	0.68	NR	NR	NR
McCrea	CheckMate 025 EQ-5D	Re 0.895 SD 0.846	0.817	NR	NR	NR
Sarfaty	CheckMate 025 FKSI-DRS	0.89	0.78	NR	NR	NR
Sarfaty	NR	NR	NR	NR	NR	NR
Barzey	MDX010–20 SF-36	CR 0.88 SD 0.80	0.52	NR	NR	NR
Curl	Literature and Expert Opinion	Re 0.88 SD 0.80	0.52	NR	−0.09	NR
Bohensky	CA209066 EQ-5D	0.828	0.798	NR	NR	NR
Oh	CheckMate 067	0.667	0.433	−0.159 IO	− 0.116 IO	− 0.115 IO
Wang	KEYNOTE 006 EQ-5D	0.83–0.86	0.78	NR	NR	NR
Miguel	KEYNOTE 006 EQ-5D	0.80	0.70	NR	NR	NR
Kohn	Literature	Re 0.88 SD first line therapy 0.80	0.52	−0.17 IO	−0.17 IO	− 0.17 IO
Meng	CheckMate 066 EQ-5D	0.78–0.80	0.71–0.73	NR	NR	NR

Note: Utility and disutility values used by the systematic review studies

*QoL* quality of life, *PF* progression free, *PD* progressive disease, *IO* immune-oncology specific, *NR* not reported, *Re* response, *SD* stable disease, *CR* complete response

Cost-effectiveness analysis can be applied to comparison of single, combination or sequential use of immunotherapies extending their applicability to cost effectiveness evaluation of immune therapies as new targets and drugs are identified. Kohn et al. used cost-effectiveness analysis to evaluate sequential versus combined ICI in melanoma [16]. The combination of PD-1/PD-L1 and anti-CTLA-4 agents increases both cost and clinical toxicity and sequential therapies starting with a PD-1/PD-L1 were more cost effective than combined therapy with a PD-1/PD-L1 and anti-CTLA-4 agents.

Once a QALY impact has been projected, the cost associated with achieving that QALY gain is expressed in cost-effectiveness analysis as the ICER. Whether that cost provides value depends on the willingness to pay (WTP) from the different perspectives of patients, providers, and public and private payers—but ICER “thresholds” have been most frequently used internationally by government payers working within a prespecified health budget. As Medicare is an entitlement program with no fixed budget, there is no federal WTP benchmark in the US. The authors selected a WTP of $100,000 as the most widely used figure in the literature, but more recent discussions have argued that a higher number is more appropriate [17]. As the authors note, raising it to $150,000/QALY would change nivolumab to being cost-effective in four of the cancers considered.

The U.S. has no standard or consensus on the appropriate WTP threshold. In its review of about-to-launch new medicines, I.C.E.R. utilizes a sensitivity analysis of $50,000 to $150,000 per QALY, but publicizes a “value-based price” based on $150,000 per QALY [8]. Their range is roughly 1 to 3 times per capita GDP (Gross Domestic Product), which was $59,500 in 2016 [18]. The lower end of range--$50,000 per QALY—was established in the early 1980s as a benchmark based on the cost of kidney dialysis [19], but it was not adjusted for quality of life nor adjusted over time for inflation or changes in the cost of care. The average annual cost of dialysis for Medicare in 2016 was $89,400. A recent systematic review suggests an average utility for dialysis patients of about 0.6. [20]. The ratio of the two is $149,000 (=$89,400/0.6), which is at I.C.E.R.’s upper bound. Clearly, every individual has a unique threshold, depending on his or her income, health preferences, and many other factors. Furthermore, different health plans and health systems would have different thresholds, as would different nations. In the United Kingdom (U.K.), the National Institute for Health and Care Excellence (NICE) recently decreased the threshold from GBL30,000 to GBL20,000 per QALY. In 2017, the GDP of the U.K. was GBL30,300 [17].

The cost-per-QALY metric is used most frequently in the decision context of health plan or formulary coverage: i..e., should access to a particular medicine be permitted (but often limited to particular subgroups of patients)? Both the recent Second U.S. Panel on Cost-effectiveness in Health and Medicine [21] and the Special Task Force on U.S. Value Frameworks of the International Society of Pharmaeconomics and Outcomes Research (ISPOR) [22] view this question from a “healthcare sector perspective.” While health gain in terms of mortality and morbidity improvements are probably what matter most to patients, both reports cite other elements that should be considered in a broader “societal perspective”, such as impacts on productivity, family members and caregivers, scientific knowledge spillovers, and uncertainty related to financial risk protection and the likelihood of benefit, among others.

While the above discussion considers cost-effectiveness of ICI for broad populations, subgroups of patients defined by clinical features may benefit to greater or lesser degree. Verma et al. point out that more focused selection of patients for treatment using host and tumor-related biomarkers could improve the effectiveness of ICI, and thereby the QALY gained, by identifying sub-populations with higher likelihood of receiving benefit or reduced toxicity. The FDA requires biomarker testing in some cancers (companion diagnostic) and recommends testing for others (complementary diagnostic). To the degree that biomarkers identify patients who are more likely to respond to ICI and lead to better clinical choices among treatment options they will increase the QALY gain of the drug. Four biomarkers are currently used to predict degrees of immunotherapy responsiveness but are not generally measured together: microsatellite instability (MSI), tumor mutation burden (TMB), PD-L1 expression and immune cell infiltrate in or around the tumor. MSI has achieved FDA approval as a biomarker that permits selection of immunotherapy with a high probability of achieving clinical benefit; however, such positivity is a relatively uncommon occurrence. MSI is a surrogate marker for deficiencies in DNA repair and similar to TMB is a surrogate marker for increased tumor related antigenicity as tumor mutations lead to neo-antigens that are potential targets of the immune system [23]. With the increased use of next-generation sequencing, TMB can be calculated based on the percentage of nonsynonymous somatic mutations per megabase sequenced and is being used to report patient TMB [24]. Yet although high TMB may increase the likelihood that an endogenous host immune response has been generated but suppressed by checkpoint mechanisms, it does not ensure that. Further, the nature of the cancer antigen in terms of protein function and immunogenicity may be more important than the number of antigen targets on the cancer cell. PD-L1 expression by immunohistochemistry can be a surrogate marker for suppression of a host immune response to the cancer and is being used to select patients for treatment for some cancers. However, the variability in testing platforms and cut off levels used to predict response, and the inconsistent predictive value of the test in different cancers has limited the utility of PD-L1 testing as recently reviewed [25]. The presence of immune cells bearing PD-L1 either surrounding or infiltrating the cancer has been suggested to predict ICI responsiveness by identifying what are referred to as hot cancers, although quantitative assessment of the degree of T cell infiltration and qualitative assessment of patterns of host immune response using immune cell biomarkers is likely to be beyond unaided human cognitive capacity Advances in the use of image recognition using artificial intelligence combined with multiple cell surface markers that can identify immune effector cell populations would enhance our understanding of the orchestration of the host immune response. A similar strategy can be pursued using gene expression analysis to predict downstream activation of immune activation pathways as has been reported for interferon gamma, a key positive and negative modulator of the immune system [26]. Greater precision in biomarkers keyed to specific aspects of regulation of the immune response and tailored to both the patient and cancer type will improve the clinical value of both ICI and emerging classes of immunotherapy of cancer.

How can we move forward an augmented QALY-based analysis to implement a more rational coverage policy that ensures access to the most advanced oncologic therapies while not simply paying whatever the market will endure irrespective of actual NHB gained? We suggest seven considerations.

First, it is generally unlikely—and certainly unclear—whether the drug prices used in these projections reflect the cost actually paid since confidential discounts are a common feature of our drug reimbursement system [17]. Greater transparency on how price is escalated as drugs move through the supply chain and specialty pharmacy distributors is needed.

Second, these are mathematical projection models based on clinical trial data often for regulatory approval: this is the best information available at launch, but it may be poor predictor of future real-world application and outcomes. This is an argument for a healthcare system that gathers post-launch real-world evidence and adjusts prices (i.e., the rewards to manufacturers) based on the actual value delivered [27].

Third, disutility values specific to immune-oncology need to be estimated through the use of PRO instruments germane to immunotherapy related adverse events. This will require either the design of new PRO instruments or modification of existing instruments currently used in oncology studies.

Fourth, value each incremental gain in longevity equally using the QALY although prolonged survival (tail of the survival curve), especially if off-treatment, might merit extra weight as is done in the ASCO and ESMO models. This approach stems from the observation that patients who have responded to ICI can have prolonged survival even off therapy suggesting that some of these patients may be cured.

Fifth, although the mean introduction price of a new ICI drug often exceeds $100,000, it is worth remembering that the marginal cost of making and distributing the medicine is far below this. Hence, we are really arguing about how much of a reward to give to the manufacturer for this innovation for a particular indication, but doing so based on very limited information at launch. The ICER value for an ICI will vary according the effectiveness of the agent in different cancer types. A good case can be made for “indication-specific pricing” (moving away from per-tablet or per-vial reimbursement) since the ICER will vary by indication (by cancer or line of therapy) at a constant price per tablet or vial [28]. Efforts are underway to implement such programs, perhaps through differential confidential discounts in manufacturer-payer contracts.

Sixth, ICERs will have limited uptake in the U.S. if their use does not acknowledge the high value our nation’s culture invests in respecting the patients’ autonomy and the physician-patient relationship embedded in shared-decision making. More specifically, utility and disutility values applied to QALY need to be refined based on PRO and clinician input. If the inputs into a QALY-based value assessment are viewed as arbitrary or dependent upon one-size-fits all determinants, we will not build the political will for change.

Seventh, oncologists must take a lead role in delineating transparent consensus and evidence-based treatment pathways. Consideration of cost-effectiveness, and thus ICERs, should be a foundational part of that evidence base.

The rapid progress in unraveling the complexity of host immune response to cancer and its constituent components such as T-regulatory cells, myeloid cells, cytokines and metabolic products of the human microbiome bodes well for patients with cancer and the physicians who study and treat these diseases. At the same time the impact of these clinical advances on the health system sustainability threatens equitable access for patients to life sustaining-treatments. Developing predictive biomarkers that identify which classes of immune-oncologic agents such as ICI provide the most benefit to a given patient will be essential and should be part of drug development. At the same time, accurately capturing adverse events of therapies using PRO will inform the overall value of a new therapeutic agent. As we create new classes of immune-oncology agents, combination or sequential therapies will be used, and teasing out the contribution of each agent to the overall outcomes of the patient will be difficult given current health economic measurement tools in use. It is not likely that assessing the value of therapy, accounting for its clinical and financial benefit and cost on both patient and societal levels, will be feasible without real-world, post drug approval evidence, which will necessitate capturing patient treatment care data outside the context of a clinical trial and require joint efforts on the part of professional societies, payers, pharmaceutical industry and government. Data will need to be collected during routine clinical care delivery and as part of quality improvement efforts and health economic research efforts using instruments such as PRO and electronic health records calibrated to collect such data without the need for extensive manual extraction and clean up as is now the norm. Payers, government agencies and industry have a critical role funding evidence collection through performance-based risk sharing arrangements [29]. Value frameworks will have limited utility unless they are able to incorporate such real-world evidence. Value frameworks are easily criticized for their imperfections, difficult to understand methodology and inability to address the perspectives of multiple stakeholders. Yet it is important to understand that these imperfect models provide the basis for rational discourse among patients, healthcare providers and systems, industry, payers and governments.

## Data Availability

Not Applicable

## Abbreviations

AEs: Adverse events
GDP: Gross domestic product
I.C.E.R.: Institute for clinical and economic review
ICER: Incremental cost-effectiveness ratio
ICI: Immune checkpoint inhibitors
MSI: Microsatellite instability
NHB: Net health benefit
PD: Progression of disease
PRO: Patient-reported outcomes
QALYs: Quality-adjusted life years
QoL: Quality of life
SD: Stable disease
TMB: Tumor mutation burden
WTP: Willingness-To-Pay
